# Evaluating the Accuracy, Completeness, and Readability of Chatbot Responses to Refractive Surgery-Related Patient Questions: A Comparative Analysis of ChatGPT and Google Gemini

**DOI:** 10.7759/cureus.88980

**Published:** 2025-07-29

**Authors:** Selva Arslan, Güldeniz Usta Küçükbezirci

**Affiliations:** 1 Department of Ophthalmology, University of Health Sciences, Sadi Konuk Training and Research Hospital, Istanbul, TUR; 2 Department of Ophthalmology, University of Health Sciences, Istanbul Training and Research Hospital, Istanbul, TUR

**Keywords:** artificial intelligence, chatgpt, google gemini, health communication, patient education, refractive surgery

## Abstract

Purpose

This study evaluates the performance of ChatGPT and Google Gemini in addressing refractive surgery-related patient questions by analysing the accuracy, completeness, and readability of their responses.

Methods

A total of 40 refractive surgery-related questions were compiled and categorized into three levels of difficulty: easy, medium, and hard. Responses from ChatGPT and Google Gemini were blinded and evaluated by two experienced ophthalmologists using standardized criteria. Accuracy was scored on a six-point Likert scale, completeness on a three-point scale, and readability using Flesch-Kincaid Grade Level, Gunning Fog Index, Simple Measure of Gobbledygook (SMOG) Index, and word count. Intra- and inter-rater reliability were assessed using intra-class correlation coefficients (ICC).

Results

Both chatbots demonstrated high intra-rater (ICC>0.75) and inter-rater reliability. Accuracy scores were similar for most questions; however, statistically significant differences were observed for harder questions, where Gemini showed slightly reduced performance compared to ChatGPT. Readability metrics revealed no significant differences between the two tools, although ChatGPT responses tended to be more detailed, while Gemini generated more concise answers. Harder questions resulted in longer and more complex responses, as indicated by higher Gunning Fog and SMOG Index scores.

Conclusions

ChatGPT and Google Gemini exhibit strong potential in patient education, with complementary strengths in accuracy, readability, and response detail. The influence of question complexity on chatbot performance highlights the need for ongoing optimization to enhance both clarity and accessibility. These findings underscore the value of integrating artificial intelligence (AI) tools into healthcare to support patient education and engagement.

## Introduction

Refractive surgery, encompassing procedures such as laser-assisted in-situ keratomileusis (LASIK) and photorefractive keratectomy (PRK), plays an essential role in correcting visual impairments like myopia, hyperopia, and astigmatism [1]. Innovations such as wavefront-guided ablation, femtosecond lasers, small incision lenticule extraction (SMILE), and implantable collamer lenses have significantly improved surgical precision, visual outcomes, and patient satisfaction [2-4]. These procedures are widely adopted by young, urban, and educated populations, and the high level of trust among ophthalmologists themselves further reflects their clinical value [5,6].

As the popularity of refractive surgery grows, so does the need for patients to access clear, accurate, and comprehensive preoperative information. Studies have demonstrated that patient education prior to surgery reduces postoperative anxiety, improves compliance, and enhances overall satisfaction [7]. Despite the abundance of online health content, its reliability is often questionable - where educational quality can vary substantially. To mitigate misinformation, there is a growing interest in leveraging artificial intelligence (AI) chatbots as supplementary educational tools.

AI-based chatbots such as ChatGPT [8] and Google Gemini [9] can provide patients with instant, personalized information using natural language processing techniques [10,11]. They offer advantages including 24/7 availability, cost-effectiveness, and high scalability, making them promising tools for patient engagement and education [12,13]. Prior research has shown that chatbots can generate medically accurate and empathetic responses across various specialties, from oncology to cardiology, and even outperform physicians in readability and communication quality under certain conditions [14-16]. However, the performance of AI chatbots within ophthalmology, particularly in the context of refractive surgery, remains underexplored.

This study aims to evaluate the performance of two widely used AI chatbots - ChatGPT [8] and Google Gemini [9] - in responding to common refractive surgery-related patient questions. Specifically, we assess their outputs in terms of accuracy, completeness, and readability. In doing so, this research contributes to the understanding of how AI-driven tools may assist in patient education within a surgical subspecialty context.

We hypothesize that large language model-based chatbots, such as ChatGPT and Google Gemini, have the potential to support refractive surgery patient education by providing accurate, complete, and readable responses. However, we anticipate that their performance may vary across these parameters depending on the complexity of patient questions.

## Materials and methods

Study design and chatbot selection

This study was designed as a comparative analysis of two large language model (LLM)-based chatbot applications: ChatGPT (version 4.0; OpenAI, San Francisco, CA, USA) accessed via https://chat.openai.com [8] and Google Gemini Advanced 1.5 Pro (Google, Mountain View, CA, USA) accessed via https://gemini.google.com [9]. Both applications were freely available for use during the study period. Default settings were applied for both chatbots without any custom configurations or system prompts. All responses were collected in December 2024 to ensure consistency and minimize potential variation due to model updates and were generated under default configurations in English, accessed from Turkey. The aim was to evaluate their performance in addressing frequently asked patient questions related to refractive surgery, with accuracy, completeness, and readability as primary evaluation parameters. To maintain impartiality, chatbot responses were anonymized and labelled only as "Chatbot A" or "Chatbot B." Evaluators were blinded to chatbot identities throughout the evaluation process.

Question set development

A set of 40 frequently asked questions regarding refractive surgery was compiled by both authors (SA and GUK) through a systematic review of publicly accessible patient education websites, including the American Academy of Ophthalmology patient education portal (https://www.aao.org/eye-health) [17], Mayo Clinic (https://www.mayoclinic.org) [18], and WebMD (https://www.webmd.com) [19]. The final questions were selected based on their clinical relevance, frequency of appearance, and clarity. Identical wording was used for each query directed to both ChatGPT and Google Gemini in December 2024, ensuring consistency in responses and minimizing variations that might result from subsequent updates or alterations in chatbot models. The full list of 40 patient questions, along with their assigned difficulty levels, is provided in Supplemental Table 1 to facilitate replication.

Prompt engineering and query standardization

To ensure consistency and reproducibility, all questions were submitted to both chatbots using identical wording without any preceding context or system prompts. Each question was entered as a new conversation to prevent influence from previous queries. Only a single response per question was analyzed. No follow-up prompts or clarification requests were used. If a chatbot requested clarification, the original question was resubmitted without modification. This approach minimized variability in responses that could arise from conversational context or prompt variations.

Evaluation criteria and scoring

Two ophthalmologists experienced in refractive surgery independently evaluated the chatbot responses. Evaluations were based on standardized scoring criteria for accuracy, completeness (Table 1), and readability.

**Table 1:** Scoring Criteria for Accuracy and Completeness Assessment

Parameter	Score	Description
Accuracy	1	Completely incorrect
	2	Mostly incorrect
	3	Partially correct but mostly inaccurate
	4	Mostly correct with minor inaccuracies
	5	Nearly completely correct
	6	Fully correct
Completeness	1	Incomplete
	2	Partially complete
	3	Fully complete

Responses rated as completely incorrect (score of 1) in accuracy were excluded from completeness assessment due to insufficient content for meaningful evaluation.

Readability was objectively measured through four linguistic metrics calculated directly from each response:

1. Flesch-Kincaid Grade Level: Indicates the U.S. school grade level required for text comprehension [20].

2. Gunning Fog Index: Reflects the years of formal education needed to understand the text [21].

3. Simple Measure of Gobbledygook (SMOG) Index: Estimates the educational level required to comprehend the content [22].

4. Word Count: Evaluates response length, as verbosity might indirectly impact readability.

The Flesch Reading Ease, Flesch-Kincaid Grade Level, and Gunning Fog Index formulas were applied solely for objective readability assessment, based on their original descriptions. As these are standard linguistic tools in the public domain, permission for use was not required, and no copyrighted content was reproduced [20-22].

Both evaluators used structured guidelines based on the Refractive Surgery Preferred Practice Pattern® by the American Academy of Ophthalmology to ensure responses aligned with current clinical standards [23]. Intra-rater reliability was assessed by re-evaluating all responses 15 days after the initial scoring, without regenerating chatbot responses due to the stochastic nature and continuous updates of LLMs. Before initiating formal evaluations, both ophthalmologist reviewers jointly reviewed the scoring criteria to ensure mutual understanding and scoring consistency, although no formal calibration or pilot testing session was conducted.

Categorization of question difficulty

To determine whether question complexity influenced chatbot performance, all 40 questions were categorized into three difficulty levels: easy, medium, and hard (Supplemental Table 1). This categorization was independently conducted by both evaluators based on clinical complexity, required medical knowledge, and necessity for multi-step reasoning.

Easy questions included basic factual inquiries or definitions, such as "What is LASIK?". Medium questions involved more detailed explanations or specific clinical knowledge, for example "How long does it take to recover from PRK?". Hard questions featured complex clinical scenarios, patient-specific considerations, or required nuanced judgment, such as "Can LASIK be performed after corneal cross-linking in keratoconus patients?".

Disagreements in classification (less than 10% of questions) were resolved through consensus. The final distribution included roughly equal numbers of questions per difficulty category (n=13-14 per category), facilitating balanced subgroup analysis.

Statistical analysis

All data were analysed using IBM SPSS Statistics for Windows, Version 23.0 (IBM Corp., Armonk, NY, USA). The distribution of variables was tested for normality using the Kolmogorov-Smirnov and Shapiro-Wilk tests. Descriptive statistics were expressed as mean±standard deviation for normally distributed continuous variables and median (minimum-maximum) for non-normally distributed variables.

For comparing chatbot performance across three difficulty levels, one-way analysis of variance (ANOVA) was used for normally distributed data, while the Kruskal-Wallis test was used for non-parametric data. Where significant differences were observed, post-hoc pairwise comparisons were conducted using the Bonferroni test (for ANOVA) or the Dunn-Bonferroni test (for Kruskal-Wallis).

Spearman’s rho correlation coefficients were calculated to examine the relationships between accuracy, completeness, and readability parameters. For normally distributed variables, Pearson’s correlation coefficients were used. Comparisons between ChatGPT and Gemini responses were conducted using independent samples t-tests (for parametric data) or Mann-Whitney U tests (for non-parametric data). A p-value less than 0.05 was considered statistically significant.

Intra-rater and inter-rater reliability were evaluated using intra-class correlation coefficients (ICC) with 95% confidence intervals to ensure consistency within and between the two ophthalmologist evaluators.

## Results

The refractive surgery-related questions analysed in this study were classified by the evaluators into three levels of difficulty - easy, medium, and hard - as shown in Table 2.

**Table 2:** Comparison of Results Based on Question Difficulty Levels ^​x^Kruskall-Wallis test; ​^y^One-way analysis of variance (ANOVA); ​^a,b^ groups with the same letter have no significant difference, median (minimum-maximum), mean±standard deviation.

	Easy	Medium	Hard	Test Statistics	p
Researcher 1 Accuracy Evaluation	5 (4-6)^a^	5 (4-6)^a^	5 (4-5)^b^	7.562	0.023^x^
Researcher 1 Completeness Evaluation	2 (1-3)	2 (1-3)	2 (1-3)	1.639	0.441^x^
Researcher 2 Accuracy Evaluation	5 (4-6)	5 (4 - 6)	5 (4-5)	5.150	0.076^x^
Researcher 2 Completeness Evaluation	2.5 (1-3)	2 (1-3)	2 (1-3)	1.054	0.591^x^
Flesch-Kincaid Grade Level	10.85 (8.7-14.6)	11.1 (8.6-14.6)	13.1 (8.8-14.6)	4.309	0.116^x​​​​​​^
Gunning Fog Index	13 (10.2-19.6)^a^	12.95 (10.2-17.4)^a^	15.7 (11.6-18.6)^b^	7.304	0.026^x^
Smog Index	13.25 (11.6-17.1)^a^	13.1 (10.8-15.9)^a^	14.5 (12.2-17)^b^	6.574	0.037^x​​​​​​^
Word Count	222.5±75.5^a,b^	274.47±80.31^a^	280.5±49.54^a^	4.991	0.009^y​​​​​​^

The reliability of the evaluation process was assessed using ICC to determine both intra-rater and inter-rater consistency. Intra-rater reliability, reflecting the consistency of evaluations by the same ophthalmologist over time, demonstrated excellent agreement, with all ICC values exceeding 0.75. Similarly, inter-rater reliability, which evaluates the agreement between the two ophthalmologists, also showed strong consistency, with ICC values indicating excellent reliability. All p-values associated with the ICC analyses were statistically significant (p < 0.05, Tables 3-5). 

**Table 3:** Comparison of Readability Parameters Across AI Tools ^x^Independent samples t-test; ^y^Mann-Whitney U test, p<0.05. SMOG: Simple Measure of Gobbledygook.

	ChatGPT	Gemini	Test Statistics	p
Flesch-Kincaid Grade Level	11.67±1.62	11.1±1.62	1.575	0.119^x^
Gunning Fog Index	13.05 (10.5-19.6)	13.1 (10.2-18.1)	813.500	0.900^y^
SMOG Index	13.3 (11.4-17.1)	13.15 (10.8-16.2)	832.500	0.758^y^
Word Count	228.88±78.75	266.7±75.83	-2.188	0.032^x^

**Table 4:** Analysis of the Relationship Between Readability Parameters Without Distinguishing AI Tools r: Spearman’s rho correlation coefficient, *p<0.05 SMOG: Simple Measure of Gobbledygook.

		Flesch-Kincaid Grade Level	Gunning Fog Index	SMOG Index	Word Count
Flesch-Kincaid Grade Level	r	-			
	p	-			
Gunning Fog Index	r	0.755	-		
	p	<0.001	-		
SMOG Index	r	0.875	0.880	-	
	p	<0.001	<0.001	-	
Word Count	r	0.026	-0.086	-0.052	-
	p	0.821	0.447	0.647	-

**Table 5:** Analysis of the Relationship Between Accuracy, Completeness, and Readability Parameters R1: Reader 1, R2: Reader 2 r: Spearman’s rho correlation coefficient, *p<0.05 SMOG: Simple Measure of Gobbledygook

	Flesch-Kincaid Grade Level r / p	Gunning Fog Index r / p	SMOG Index r / p	Word Count r / p
ChatGPT				
R1 Accuracy	-0.205 / 0.204	-0.182 / 0.262	-0.239 / 0.137	0.019 / 0.909
R1 Completeness	0.114 / 0.485	-0.088 / 0.588	0.003 / 0.985	0.278 / 0.083
R2 Accuracy	-0.254 / 0.114	-0.340 / 0.032*	-0.308 / 0.053	-0.081 / 0.617
R2 Completeness	-0.126 / 0.439	-0.391 / 0.013*	-0.256 / 0.110	0.216 / 0.181
Gemini				
R1 Accuracy	-0.052 / 0.750	-0.108 / 0.508	-0.154 / 0.343	0.069 / 0.671
R1 Completeness	-0.040 / 0.805	-0.236 / 0.143	-0.166 / 0.305	0.139 / 0.392
R2 Accuracy	-0.111 / 0.497	-0.199 / 0.218	-0.192 / 0.236	0.015 / 0.929
R2 Completeness	-0.017 / 0.915	-0.142 / 0.381	-0.044 / 0.788	0.189 / 0.244

While the number of evaluators was limited to two, the consistently high ICC values indicated strong intra- and inter-rater agreement, supporting the reliability of the evaluation process (Supplemental Table 2).

## Discussion

Refractive surgery continues to evolve as a prominent field in ophthalmology, where the availability of reliable and understandable patient information plays a vital role. This study assessed how two AI-based chatbot platforms - ChatGPT and Google Gemini - respond to frequently asked questions on refractive surgery, using accuracy, completeness, and readability as key evaluation metrics.

Although both chatbots performed similarly in many areas, some distinctions emerged. Notably, Gemini produced longer responses, while ChatGPT showed slightly stronger performance in certain accuracy-related evaluations, particularly with straightforward questions. These differences suggest that AI-generated answers may benefit from adjustments based on the complexity of the content being addressed.

In a comparable study by Chen et al., GPT-4 responses to cancer-related patient questions were found to be highly readable and well-structured [24]. Similarly, our study showed that both AI tools provided responses with consistent readability scores. While Gemini tended to generate more detailed answers, its output remained accessible, reinforcing its suitability for educational purposes.

Burnette et al. evaluated chatbot performance in a different clinical domain-immune-related adverse events and found that ChatGPT achieved high scores for both accuracy and completeness [25]. Our observations align with these results, especially for questions that required factual recall. However, as question complexity increased, accuracy levels declined, pointing to limitations that still exist in managing nuanced or context-dependent queries. Although our evaluation focused primarily on quantitative metrics such as accuracy and readability, both evaluators informally noted that some responses - while factually correct - lacked sufficient clinical nuance or contextual appropriateness. These qualitative impressions underscore the importance of not only factual precision but also the ability to convey medically relevant and patient-centered explanations, particularly in more complex scenarios.

A study by Adithya et al. compared the readability of AI-generated brochures and reported similar outcomes for ChatGPT and Gemini, with variations in verbosity and structure [26]. Our data showed a comparable pattern, where ChatGPT often gave more elaborated responses, while Gemini's outputs leaned toward conciseness. Despite these stylistic differences, no significant variation was observed in standard readability indices such as Flesch-Kincaid or SMOG.

Performance differences based on question complexity were evident in our analysis and supported by other research. Baglivo et al. noted that AI models handle direct questions more accurately than complex or negatively phrased ones [27]. Similarly, Friederichs et al. found that ChatGPT's accuracy decreased as question difficulty increased [28]. Our results reflect this trend, as harder questions received lower accuracy scores from evaluators.

Yu et al. also examined ChatGPT's capabilities on open-ended medical questions and reported a notable drop in performance for case-based scenarios [29]. In our study, responses to complex refractive surgery questions showed similar challenges, including reduced accuracy and increased linguistic complexity, which may hinder comprehension for patients with limited health literacy.

Uppalapati and Nag compared multiple AI models and found that while ChatGPT consistently offered relevant and accurate responses, other models such as Claude AI performed better in completeness [30]. While our work did not include Claude, we observed that Gemini also generated detailed answers, suggesting that newer AI tools may complement existing ones depending on content needs.

A distinctive observation in our study was the effect of question difficulty on readability measures. Gunning Fog and SMOG indices increased for harder questions, indicating that chatbot-generated responses became more complex as the question content deepened. This could pose challenges for patients with lower reading proficiency, especially when clarity is essential for informed decision-making. This pattern may also reflect limitations in domain-specific training data, as large language models may have had less exposure to detailed refractive surgery content compared to more general medical topics. Such gaps in training can contribute to underperformance, particularly in complex or highly specialized queries.

In clinical ophthalmology, chatbot platforms may be integrated into patient care workflows as supplementary tools to assist with preoperative education, deliver standardized responses to frequently asked questions, and support postoperative guidance via institutional portals or mobile health applications. Such integration may enhance efficiency, reinforce clinician-patient communication, and empower patients to engage more actively in their care.

In summary, the results demonstrate that AI chatbots like ChatGPT and Gemini are promising tools for supporting patient education in refractive surgery. Their outputs are generally accurate and readable, especially for simpler inquiries. However, there is room for improvement when it comes to adapting responses for more complex questions and ensuring accessibility across varying literacy levels. As AI technology progresses, refining these systems to meet the specific needs of medical communication will remain a priority.

Limitations

This study has several limitations that should be considered when interpreting the results. First, while our evaluation primarily focused on quantitative measures such as accuracy, completeness, and readability, the study did not include a qualitative assessment of the reasoning or explanation depth provided by the chatbots. Future studies should also investigate whether chatbot responses convey adequate clinical nuance and reassurance, as quantitative accuracy alone may not guarantee appropriateness or usefulness from a patient’s perspective. Although quantitative scoring offers objectivity and standardization, future studies may benefit from including structured qualitative analyses to better capture the nuance of response quality.

Another consideration involves the limited sample size of questions (n=40) and the number of evaluators (two ophthalmologists). This restricted sampling may constrain the generalizability and robustness of the findings despite high inter-rater reliability among experienced refractive surgeons. Future studies should include larger and more diverse question sets, as well as broader evaluator panels - encompassing clinicians, patients, and laypersons - to enhance external validity and better assess chatbot performance. Additionally, the questions used in this study were sourced from real-world patient inquiries but may not fully encompass the breadth of refractive surgery-related concerns. Expanding the scope and diversity of question sets in future research may also strengthen the reliability and applicability of performance assessments.

Additionally, this study did not include input from patients or laypersons, whose perspectives on readability, trustworthiness, and usability are essential for evaluating real-world applicability. While objective readability metrics were employed, the exclusive use of ophthalmologist evaluators may not fully capture how patients interpret or trust chatbot-generated responses. This may particularly affect real-world integration, where patient comprehension and confidence in AI-provided information are critical. Future research should incorporate direct feedback from refractive surgery patients and non-expert users to better assess perceived clarity, trust, and utility in clinical communication contexts.

Furthermore, the exclusive use of text-based prompts in this study may not fully reflect the multimodal nature of clinical communication in ophthalmology, which often involves image interpretation (e.g., slit-lamp photographs, topographies, optical coherence tomography (OCT) scans). As such, chatbot performance in scenarios requiring visual data analysis remains unassessed. Future research should explore the integration of image- or video-based queries to evaluate chatbot capabilities in handling multimodal clinical tasks more representative of real-world practice.

The dynamic and version-specific nature of AI models presents another inherent challenge. Both ChatGPT and Google Gemini are continually updated, which may affect the reproducibility of results over time. To minimize this, we evaluated all chatbot responses during a defined time period and chose not to re-query the models after updates to maintain internal consistency. While this decision improves reliability within the study design, it limits the temporal generalizability of the findings.

Moreover, the stochastic nature of large language models - where slight variations in outputs may occur for identical prompts - was not addressed by running multiple response iterations. Future research may incorporate repeated queries to better assess response variability and consistency.

We also acknowledge that the exclusion of image-based or multimodal queries - common in ophthalmology - limits the clinical relevance of chatbot responses in scenarios where visual data interpretation is required.

Additionally, the influence of prompt structure and phrasing on chatbot performance, often referred to as contextual dependence, was not varied in this study. Although all questions were standardized and identical across models to ensure a fair comparison, exploring variations in prompt wording could offer insights into the robustness of AI tools in more naturalistic settings. Refractive surgery is a domain where much of the surgical technology, clinical data, and outcome algorithms are proprietary or unpublished. This closed-source nature may impact both the quality and completeness of AI-generated educational content, as highlighted by Jiao et al. [31]. Because foundational clinical datasets and surgical protocols are often inaccessible to both the public and developers, AI models may be trained on incomplete or outdated information, which could limit their performance, particularly in highly specialized fields like refractive surgery.

Lastly, this study focused primarily on quantitative evaluation metrics such as accuracy, completeness, and readability. While these measures provide objective insight into chatbot performance, they do not capture the clinical nuance or appropriateness of responses in the context of patient education. Quantitative accuracy does not necessarily equate to clinical relevance or reassurance. Future studies should incorporate structured qualitative content analysis to evaluate the depth, tone, and contextualization of responses - factors that are especially important in patient-facing applications.

Despite these limitations, this study offers valuable insights into the real-world performance of AI chatbots in a focused clinical context. It provides a structured and reproducible framework that can serve as a foundation for future investigations, especially those integrating both quantitative and qualitative methods.

Future work should not only examine the accuracy of AI chatbot responses but also assess the quality and validity of the sources cited, as some models are beginning to include references. Expert evaluation of these citations may help improve transparency, traceability, and credibility in AI-driven patient education.

## Conclusions

This study presents a structured evaluation of two advanced AI chatbots - ChatGPT and Google Gemini - in their ability to answer common refractive surgery-related patient questions. By assessing accuracy, completeness, and readability, the research offers insights into the practical utility and current limitations of AI tools in patient education. The findings demonstrate that both chatbots provide responses of generally high quality, with comparable performance across most parameters. However, notable differences emerged in specific areas, such as word count and performance on more complex questions.

While ChatGPT demonstrated slightly higher accuracy in certain scenarios, Gemini produced longer and more elaborate responses, underscoring different strengths in information delivery. Readability metrics, particularly the Gunning Fog and SMOG indices, varied significantly based on question difficulty, highlighting the influence of content complexity on linguistic accessibility. These outcomes suggest that the design and optimization of chatbot responses should consider both the nature of the medical topic and the intended patient audience.

As AI chatbots continue to evolve and become more integrated into healthcare communication, ensuring balanced, accurate, and accessible responses will be critical. The results of this study support the responsible use of AI tools as supplementary resources in patient education, while also emphasizing the ongoing need for refinement, validation, and oversight in clinical communication contexts.

## Appendices

**Table 6:** Supplemental Table 1: Questions included in the study with categorization into three difficulty levels

Question	Difficulty
How does LASIK work?	Medium
What vision conditions does LASIK correct?	Easy
How long does LASIK surgery last?	Easy
What is the difference between LASIK and SMILE?	Medium
What is the difference between LASIK and PRK?	Medium
Can LASIK get rid of my reading glasses?	Easy
I have astigmatism. Can I have refractive surgery?	Easy
Is there a limit as to how much nearsightedness, farsightedness, or astigmatism one can have to be a candidate for refractive surgery?	Hard
Are both eyes treated the same day with refractive surgery?	Easy
What is done for the discomfort after PRK?	Easy
What kind of vision can I expect a month after PRK or LASIK?	Medium
Anything special I need to do if I wear contact lenses, before the refractive surgery?	Easy
Am I too old to have LASIK?	Easy
Does refractive surgery hurt?	Easy
What if I blink or move during the refractive surgery procedure?	Medium
How soon after the refractive surgery will I be able to see?	Medium
When will I be able to drive after refractive surgery?	Easy
What are the possible complications of refractive surgery?	Medium
I’ve heard that the laser surgery causes night vision problems. Is this true?	Medium
Will I have to limit my activities after refractive surgery?	Easy
How soon can I use eye make-up after laser vision correction?	Easy
How much time from work will I have to miss after refractive surgery?	Easy
Will I feel the lens implant of refractive surgery?	Medium
Can you guarantee 20/20 vision with refractive surgery?	Medium
I have "dry eyes." Will this affect my LASIK surgery?	Medium
What is monovision?	Hard
Can there be a problem with my eyes 20 years from now because I had LASIK?	Medium
If I had refractive surgery, can I have cataract surgery if I need it in the future?	Hard
Can I play sports right after the refractive surgery procedure?	Easy
Can I get water in my eyes after refractive surgery?	Easy
What are enhancements (touch-ups) after refractive surgery?	Hard
Is it possible that my vision could be worse than before after refractive surgery? Could my vision gradually decline?	Easy
Is there a maximum graduation limit to be considered suitable for refractive surgery?	Medium
Is it possible to obtain a zero diopter result through refractive surgery?	Easy
Can pregnancy change the outcome of refractive surgery?	Medium
For which cases are phakic intraocular lenses (ICL) indicated?	Easy
Is the intraocular lens, used in refractive surgery, visible?	Easy
Is displacement of the implanted lens, used in refractive surgery, possible?	Medium
Are you awake for laser surgery?	Easy
How long is it blurry after laser surgery?	Easy

**Table 7:** Supplemental Table 2: Analysis of Differences Between Researchers *Wilcoxon test, median (minimum-maximum), *p<0.05

	ChatGPT	Gemini
Researcher 1 Accuracy	5 (4-6)	5 (4-6)
Researcher 2 Accuracy	5 (4 - 6)	5 (4-6)
Test statistics	-1.155	-2.496
p	0.248	0.013*
Researcher 1 Completeness	2.5 (1-3)	2 (1-3)
Researcher 2 Completeness	3 (1-3)	2 (1-3)
Test statistics	-1.890	-1.265
p	0.059	0.206
